# Prognostic value of chest computer tomography combined with serum platelet count, c-reactive protein levels and oxygenation index in severe community-acquired pneumonia

**DOI:** 10.12669/pjms.41.2.10405

**Published:** 2025-02

**Authors:** Yun Wu, Sijie Xu, Yi Xia

**Affiliations:** 1 Yun Wu, Intensive care unit, The 72^nd^ Group Army Hospital of PLA, Huzhou, Zhejiang Province 313000, P.R. China; 2 Sijie Xu, Department of Radiology, Huzhou Central Hospital, Affiliated Central Hospital HuZhou University, Huzhou, Zhejiang Province 313000, P.R. China; 3 Yi Xia, Department of Radiology, Huzhou Hospital, Huzhou, Zhejiang Province 313000, P.R. China

**Keywords:** C-reactive protein, Chest CT, Oxygenation index, Platelet count, Severe community-acquired pneumonia

## Abstract

**Objective:**

To investigate the prognostic value of chest computed tomography (CT), platelet count (PLT), serum C-reactive protein (CRP) level, and oxygenation index (OI) in patients with severe community-acquired pneumonia (CAP).

**Methods:**

We conducted a retrospective analysis of clinical data collected from 226 patients with CAP who received treatment in Huzhou Central Hospital from February 2022 to November 2023. Patients were divided into two groups based on pneumonia severity: Severe group (patients with severe CAP, n=113) and Typical group (patients with typical pneumonia, n=113). Differences in CT score, PLT, CRP, and OI levels between the two groups were analyzed, as well as the prognostic value of the combined CT score, PLT, CRP, and OI levels in severe CAP.

**Results:**

The CT Score and CRP level in the Severe group were significantly higher than those in the Typical group, whereas PLT and OI were significantly lower (*P*<0.05). Of 113 patients with severe pneumonia, 42 died and 71 survived. The CT Score and CRP level in the death group were significantly higher, whereas PLT and OI were lower compared to the survival group (*P*<0.05). The area under the ROC curve of the combined CT Score, PLT, CRP, OI for the prediction of death in patients with severe CAP was 0.970, sensitivity was 85.7, and specificity was 93.0, which was higher than that of each index alone.

**Conclusions:**

The combined chest CT, PLT, CRP, and OI have high prognostic value for severe CAP.

## INTRODUCTION

Severe community-acquired pneumonia (CAP), the most life-threatening form of CAP, is characterized by intensive care unit admission and high mortality rate and significant morbidity.^1^,^2^ Studies have shown that the progression of severe CAP is accompanied by increased secretion of inflammatory mediators, which can aggravate systemic inflammatory responses, lead to complications, such as meningitis, empyema, and multiple organ dysfunction,^3^,^4^ and profoundly affect quality of life and health of patients.^2^–^4^ It is noticeable that early accurate and effective assessment of the prognosis of patients with severe CAP to guide clinical treatment is of great significance for the improvement of prognosis.^2^–^5^

Chest computer tomography (CT) has been shown to have good performance in describing the pulmonary involvement, severity of oxygenation disorder, and can dynamically observe lung lesions through CT quantitative score.^6^,^7^ Biochemical index detection, such as platelet count (PLT) and serum levels of c-reactive protein (CRP), are also commonly used diagnostic methods for cases of severe CAP as they play a crucial role in infection and inflammation.^8^,^9^ Oxygenation index (OI), defined as the reciprocal of PaO_2_/FiO_2_ ratio times mean airway pressure, is an important evaluation index for assessing intrapulmonary shunt and mean airway pressure. It is closely related to the degree of lung injury, and therefore, is used for the diagnosis and evaluation of severe CAP.^10^

Chest CT, PLT, CRP, and OI are commonly used to diagnose CAP, but their application in predicting the prognosis of CAP is not obvious. Although the prognostic value of these indicators in patients with CAP has been recently studied, data on their prognostic predictive ability in severe CAP remain scarce.^11^-^13^ Moreover, there is currently no data on the combined role of these indicators in predicting the prognosis of severe CAP. As such, this retrospective study aimed to assess the prognostic values of chest CT, PLT, serum CRP level, and OI in patients with severe CAP, especially when applied in combination.

## METHODS

We conducted a retrospective analysis of clinical data of patients with CAP who received treatment in Huzhou Central Hospital from February 2022 to November 2023.

### Inclusion Criteria:

Patients were included if;

- Diagnosed with typical CAP or severe CAP;^14^
- Age >18 years; 3) underwent complete chest CT, RP, PLT, and OI examinations

### Exclusion Criteria:

- Patients were excluded if
- With other respiratory system diseases;
- With malignant tumors;
- With blood system diseases;
- With other infectious diseases;
- With immune system diseases;

Received immunosuppressive agents or other relevant treatments before inclusion in the study. Patients were divided into two groups based on pneumonia severity: Severe group (patients with severe CAP) and Typical group (patients with typical CAP).

### Ethics approval:

This study was approved by the Medical Ethics Committee of the Huzhou Central Hospital (2023-LW-17, Date: December 13^th^ 2023). Informed consent was waived because of the observational and retrospective nature of the study.

### Diagnosis of typical CAP and severe CAP:

The diagnosis of typical CAP and severe CAP was based on the CAP guideline from the 2007 Infectious Diseases Society of America/American Thoracic Society.^15^

### Typical CAP:

The diagnosis of typical CAP was based on a history of dyspnea, cough, pleuritic pain, or acute functional or cognitive decline, as well as abnormal vital signs (eg, tachycardia, fever) and findings of pulmonary examination.^15^,^16^ The diagnosis should be confirmed by chest radiography or ultrasonography.

### Severe CAP:

The diagnosis of severe CAP should include ≥ one major criterio or ≥ three minor criteria listed below:^15^

- Major criteria:
- Respiratory failure requiring mechanical ventilation;
- Septic shock requiring vasopressors.

### Minor criteria:

Respiratory rate ≥30 breaths/min;, PaO_2_/FiO_2_≤250; .Multilobar infiltrates; .Confusion/disorientation; Blood urea nitrogen ≥20 mg/dL; White blood cell count < 4000 cells/μl; platelet count < 100,000 cells/μl; Core temperature < 36°C; hypotension requiring aggressive fluid resuscitation.

### Collected indicators:

### Basic information:

Sex, age, body mass index (BMI), and disease course.

### Chest CT score:

**Fig.1:**
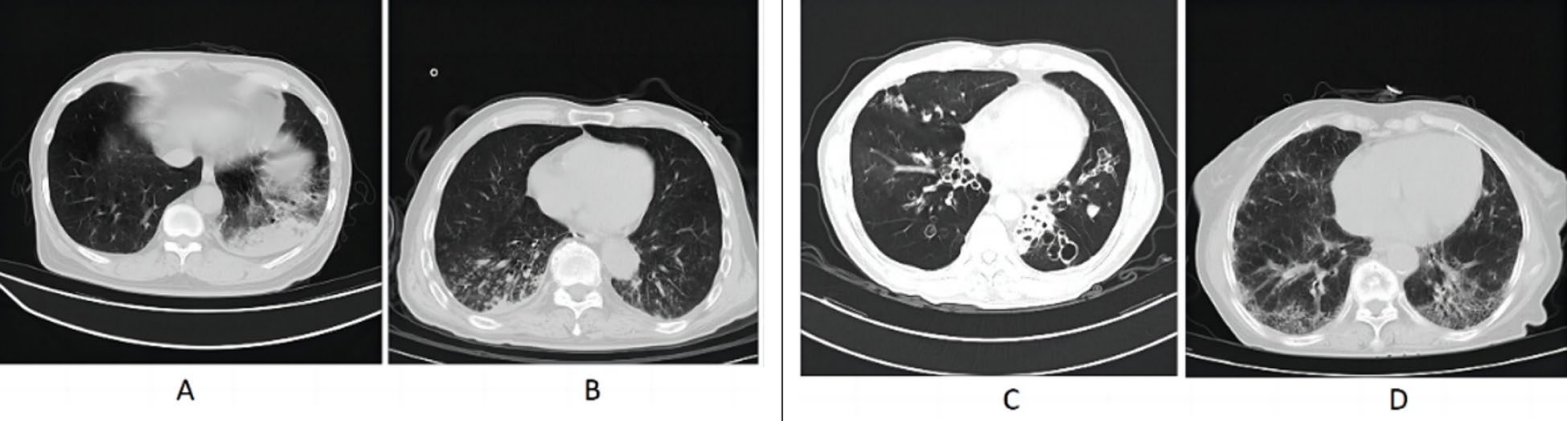
Chest CT images of patients with severe CAP. A: Male, 56 years old, with a large high-density shadow in the lower lobe of the left lung; B: Male, 62 years old, with multiple patchy and nodular high-density shadows in both lungs; C: Female, 63 years old, with extensive bronchiectasis and infection in both lungs; D: Female, 59 years old, with multiple patchy and grid like high-density shadows in both lungs.

Patients underwent chest CT examination within 48 hours after admission using Siemens Somatom Definition 64 slice spiral CT system. Patients were instructed to remain supine on the bed and was instructed to inhale and then hold his/her breath during the examination to complete the acquisition of the image results. The scanning range was from the lung apex to the level of the diaphragm. Scanning parameters were set as follows: layer thickness, 5-mm; reconstructed layer thickness: 1.25 mm, pitch, 1.20; voltage: 120 kV, current: 240mA. Chest CT results were analyzed by two experienced radiologists. Based on the results of the examination, the CT of the lungs was divided into five regions based on their anatomical location. According to the involvement of various lung lobe lesions, the Likert six levels scoring method (0-5 points) was used for evaluation. No involvement was scored as 0; 1 point for lesions involving less than 10%; 10%≤lesion involvement range < 26% is 2 points; 26% ≤ lesion involvement range < 50% is three points; 50%≤lesion involvement range < 76% is four points; five points for lesions involving a range of ≥ 76%. The total score of five regions was 0-25 points, with higher scores indicating more severe symptoms (Fig.1).

### Biochemical index collection:

Patients underwent fasting blood collection on the day following admission to the hospital. CRP levels were measured using an AU480 fully automated biochemical analyzer (Beckman Coulter, USA) and a matching reagent kit, using immunoassay turbidimetry. The PLT was measured using a fully automated blood analyzer (Mindray, China) and a matching reagent kit using the resistance method. A blood gas analyzer (Mindray, China) was used to measure OI.

Patients with severe CAP were subsequently divided into survival group and death group based on their survival status at 28^th^ day.

### Statistical Analysis:

All data analyses were done using the SPSS software (version 25.0; IBM Corp, Armonk, NY, USA). Quantitative data were expressed as mean ± standard deviation, and independent sample t-test was used for comparison between groups. Counting data were expressed as n (%), and Chi-square test was used for comparison between groups. Receiver operating characteristic (ROC) curve analysis was used to assess the prognostic value of chest CT score, PLT, CRP, OI, and combined examination in patients with severe CAP. P<0.05 was considered statistically significant.

## RESULTS

A total of 298 patients were screened for eligibility, and 43 patients were excluded. We ended up including 113 patients with severe CAP and 113 patients with typical CAP in this study. There was no significant difference in baseline data between the two groups (P>0.05) Table-I. CT score and CRP levels of patients in the Severe group were higher than those in the Typical group, while PLT and OI were significantly lower in the Severe group than in the Typical group (P<0.05) Table-II.

**Table-I:** Comparison of baseline data between two groups of patients.

Group	Gender (male/female)	Age (year)	BMI (kg/m²)	Course of disease (day)
Severe group (n=113)	72/41	55.93±8.53	23.40±2.64	4.78±1.94
Typical group (n=113)	63/51	54.08±7.92	22.81±2.90	4.45±2.08
*χ^2^/t*	1.683	1.694	1.607	1.242
*P*	0.195	0.092	0.109	0.216

**Table-II:** Comparison of clinical indicator levels between the Severe group and the Typical group

Group	n	CT score	PLT (×10^9^/L)	CRP (mg/L)	OI (mmHg)
Severe group	113	10.07±3.07	181.68±40.31	28.00±6.22	157.96±36.24
Typical group	113	6.53±1.79	200.63±53.54	23.25±5.95	188.39±41.94
*t*		10.644	-3.014	5.888	-5.851
*P*		<0.001	0.003	<0.001	<0.001

**Fig.2:**
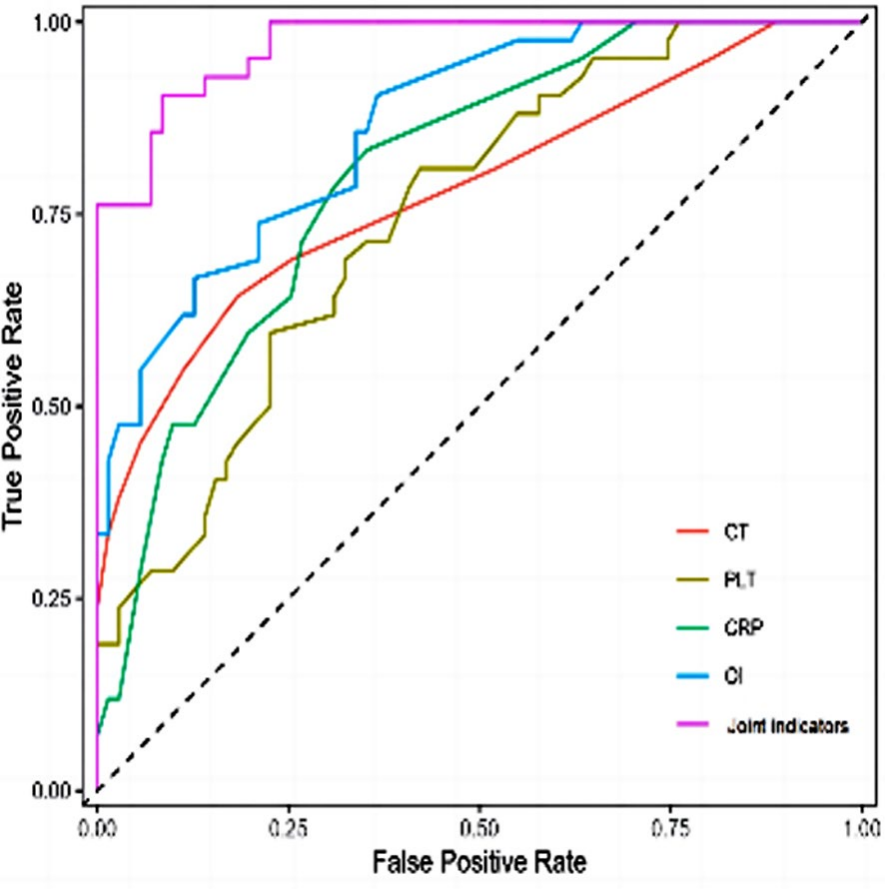
ROC curve of CT score, PLT, CRP, OI, and combined prediction of prognosis in patients with severe CAP.

Of 113 patients with severe CAP, 42 died and 71 survived. The CT score and CRP level of the death group were significantly higher, whereas PLT and OI were significantly lower compared to the survival group (P<0.05) Table-III. The area under the ROC curve of CT score, PLT, CRP, and OI combined for predicting death in patients with severe CAP was 0.970, with a sensitivity of 85.7 and a specificity of 93.0, higher than the individual value of each indicator (Table-IV, Fig.2).

**Table-III:** Comparison of CT scores, PLT, CRP, and OI levels among patients with different prognoses.

Variable	Death group (n=42)	Survival group (n=71)	χ2/t	P
CT score	12.19±3.37	8.82±2.03	-5.882	<0.001
PLT (×10^9^/L)	159.86±29.57	194.59±40.41	4.851	0.039
CRP (mg/L)	32.07±6.45	25.59±5.74	-6.179	<0.001
OI (mmHg)	130.60±25.65	174.15±31.64	-7.568	<0.001

**Table-IV:** Diagnostic efficacy of CT score, PLT, CRP, OI, and their combination in predicting the prognosis of patients with severe CAP.

Variable	AUC	Sensitivity	Specificity	Cut-off	Youden	95%CI
CT score	0.782	54.8%	88.7%	10.511	0.435	0.690~0.873
PLT	0.749	45.2%	81.7%	185.994	0.269	0.659~0.839
CRP	0.803	59.5%	80.3%	26.513	0.398	0.723~0.883
OI	0.866	61.9%	88.7%	145.997	0.506	0.800~0.931
Combined indicators	0.970	85.7%	93.0%	-8.316	0.787	0.946~0.995

Combined indicators, the combination of CT Score, PLT, CRP, and OI.

## DISCUSSION

The treatment of severe pneumonia is mainly based on the comprehensive analysis of laboratory tests, pathogenetic tests and imaging results, so as to carry out symptomatic treatment. Finding specific indicators that reflect changes in the condition to assess the prognosis of the disease and to take proactive countermeasures is the key to improving the prognosis. Our study demonstrated the association of PLT and CRP with the severity and prognosis of pneumonia. Our results are in agreement with previous reports.^13^,^17^ Yardımcı et al.^18^ investigated the expression of PLT and other factors in pneumonia patients, and showed that compared to pneumonia patients in the general ward, baseline and 3-day PLT in pneumonia patients in the intensive care unit were significantly reduced, and there was a close correlation between PLT levels and pneumonia mortality rate. Additionally, PLT was shown to play an important role in inflammatory response and immune regulation in the body. Barak-Corren Y et al.^19^ found that CRP is an acute-phase reactive protein generated by the liver that can exert various immune functions such as phagocytosis and regulation. Their study demonstrated that while under normal physiological conditions serum content of CRP is low, it abnormally increase in cases of bacterial infection, reaching its peak within 36-50 hours. Additionally, CRP levels in pneumonia patients are abnormally elevated. By dynamically monitoring their levels, disease progression and prognosis can be predicted, thereby providing important guidance for disease treatment. Gunaratnam et al.^20^ investigated the diagnostic value of CRP in pneumonia, and showed that its sensitivity and specificity were 70.0% and 64.0%, respectively, which were superior to indicators such as WBC count. However, there is still significant room for improvement in the diagnostic sensitivity and specificity of CRP.

Our study also demonstrated that OI has high prognostic value in patients with severe CAP. Similarly, Ergan et al.^21^ pointed out that OI is an important indicator for clinical evaluation of lung tissue ventilation and functional status, and its clinical use can avoid inaccurate assessment of lung tissue damage caused by high arterial PaO_2_ concentration due to inhalation of oxygen, and accurately assess the degree of lung tissue damage and organ tissue hypoxia. Additionally, continuous dynamic monitoring of OI and targeted treatment based on it can help reduce body oxygen consumption and regulate microcirculation status.

However, while numerous studies explored the application value of chest CT, PLT, CRP, and OI in severe CAP, their combined prognostic value was not assessed.^18^-^21^ Therefore, the results and conclusions of our study provide a reference for the diagnosis and evaluation of severe CAP. This study conducted a statistical analysis on the prognosis of patients with severe CAP, and detected significant association in CT scores, and PLT, CRP, and OI levels with the prognosis. The results of ROC curves also indicated that chest CT, PLT, CRP, and OI have important application values in predicting the prognosis of severe CAP.^21^-^23^ Our results have direct application value for clinical practice, as using these indicators allows to implement corresponding interventions to minimize adverse disease outcomes and improve disease survival rates.^23^,^24^

### Limitations:

First, this is a single-center retrospective study. Second, CT indicators may be influenced by human or technical factors. Third, the study investigated the prognostic value of the combined CT Score, PLT, CRP, and OI, but did not further explain to what extent it improved the diagnostic sensitivity and specificity compared with other approaches. Further comparison should be conducted in future research to verify our conclusions.

## CONCLUSION

The combined chest CT, PLT, CRP, and OI have high prognostic value for severe CAP. Targeted interventions that are based on the CT scores, and the levels of PLT, CRP, and OI can be performed in clinical practice to ensure good outcomes in patients with severe CAP.

### Authors’ Contributions:

**SX** conceived and designed the study. Literature search. **YW, SX and YX** collected the data and performed the analysis. Critical review. **YW and SX** was involved in the writing of the manuscript, literature search. All authors have read , approved the final manuscript and are responsible for the integrity of the study.
